# Dataset on constructing colloidal nanoparticles into dry nano-micro-particle (NMP) powders with Nanoscale Magnetic, Plasmonic and Catalytic Functionalities

**DOI:** 10.1016/j.dib.2019.103928

**Published:** 2019-04-18

**Authors:** Qinglu Chen, Ziwei Ye, Chunchun Li, Hannah McCabe, Jessica Kelly, Yikai Xu, Steven E.J. Bell

**Affiliations:** School of Chemistry & Chemical Engineering, Queen's University Belfast, BT9 5AG, UK

## Abstract

The data presented in this article is related to the research article entitled “A One-Pot Method for Building Colloidal Nanoparticles into Bulk Dry Powders with Nanoscale Magnetic, Plasmonic and Catalytic Functionalities” (Ye et al., 2019). The data shows the hydrophobicity of the nanoparticle (NP) building blocks used for constructing NMPs obtained through contact angle measurements, along with the effect of NP hydrophobicity on the stability of the parent Pickering emulsions. SEM data of the morphology of NMPs is presented. Finally, a mathematical model is presented to predict the average diameter of NMPs produced via different experimental parameters.

**Table:** Specifications Table

Subject area	*Chemistry*
More specific subject area	*Surface-enhanced Raman spectroscopy, Nanostructured materials*
Type of data	*Image, text file, figure*
How data was acquired	*SMZ800 N Optical Microscope, Quanta FEG SEM, First Tech Angstroms FTA125 Goniometer- Contact Angle & Surface Tension Machine*
Data format	*Raw, analyzed*
Experimental factors	*Contact angle measurements were performed by adding* 1 μL *of water onto the sample using an automated system and then analyzed* via *computer program. SEM used a Quanta FEG 250 at an accelerating voltage of* 30 kV *under high chamber pressure (8* × *10*^*−5*^*mbar) with standard SEM copper tape or carbon tape as background.*
Experimental features	*Nano-micro-particles were prepared using an emulsion-templated method*
Data source location	*Belfast, UK*
Data accessibility	*Data is with this article*
Related research article	*Z. Ye, C. Li, N. Skillen, Y. Xu, H. McCabe, J. Kelly, P. Robertson, S. E. J. Bell, A One-Pot Method for Building Colloidal Nanoparticles into Bulk Dry Powders with Nanoscale Magnetic, Plasmonic and Catalytic Functionalities, Appl. Mater. Today, 15 (2019) 398–404.*

**Table:** 

**Value of the data** • The dataset provides future reference for the hydrophobicity of the NP-building blocks required for generating stable Pickering emulsions using promoters. • The dataset provides future reference for calculating the surface-coverage of capping agents on NP assemblies fabricated using promoters. • The dataset provides future reference for fabricating NMPs of various diameters.

## 1 Data

**Fig. 1:**
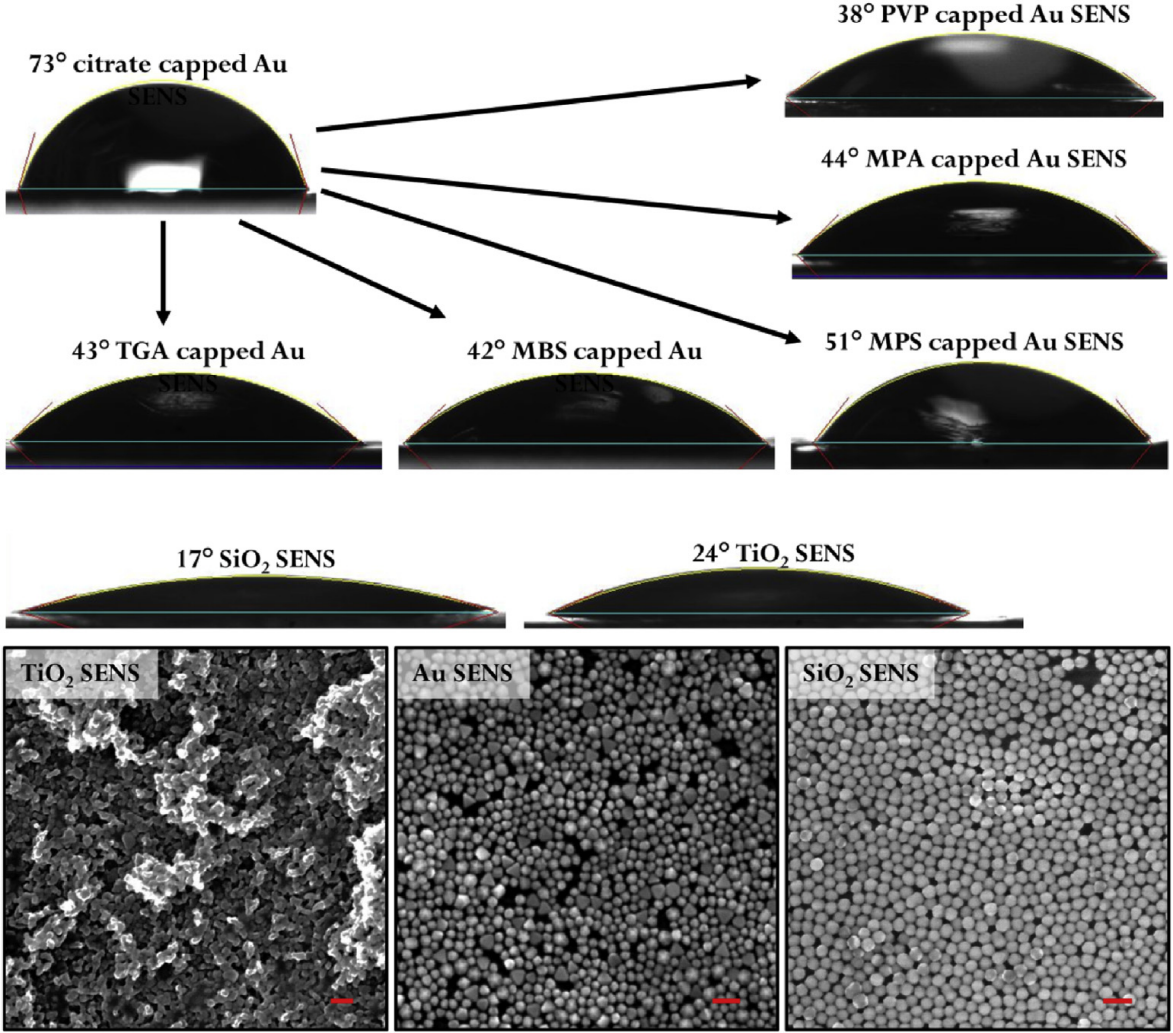
Contact angle data for SENS carrying different types of NP layers or the same type of NP with different types of molecular capping. SEM data of the surface of typical SENS films. All scale bars in SEM images correspond to 100 nm. All values shown are based on automated droplet profile fitting and are accurate within ± 5°.

**Fig. 2:**
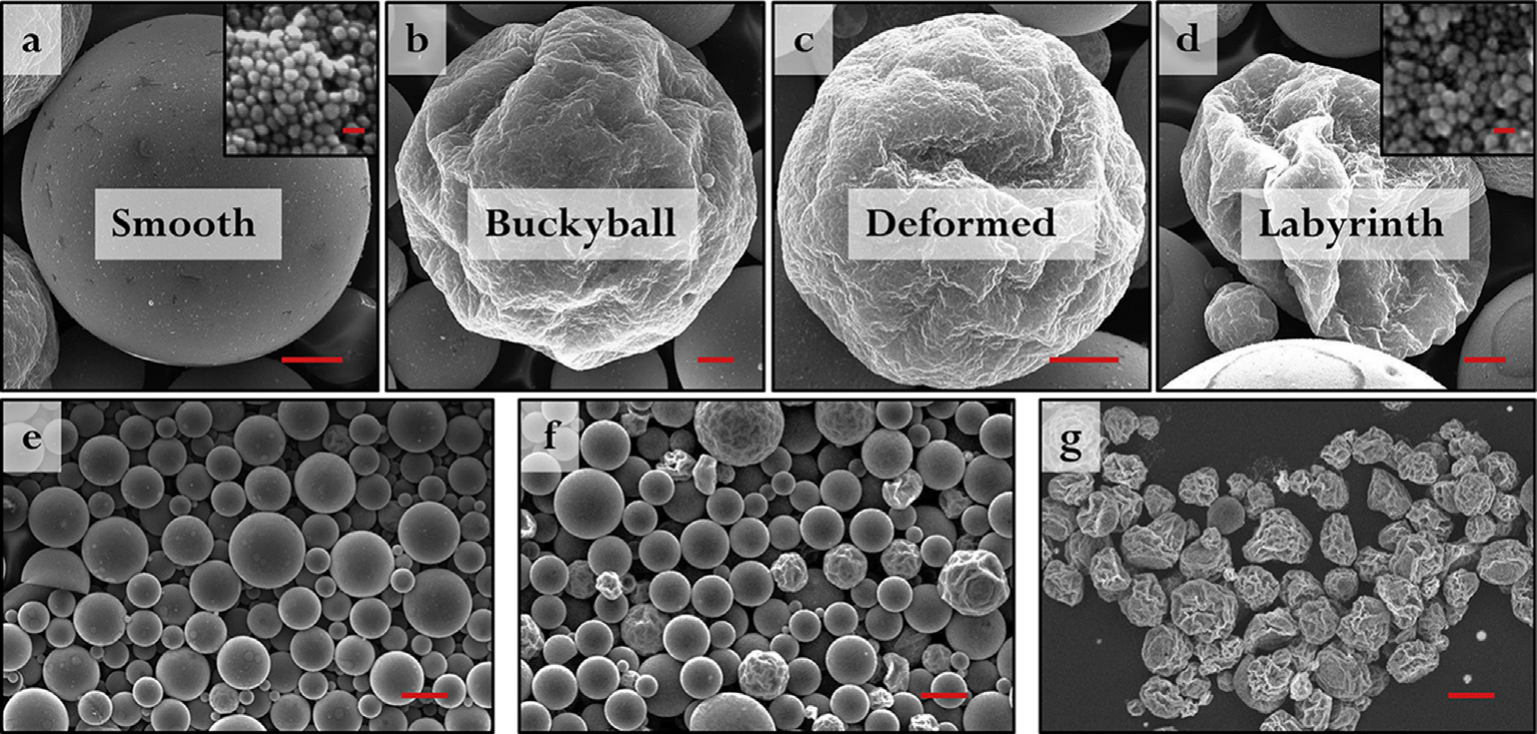
SEM data of NMPs observed at different stage of buckling caused by DCM evaporation: (a) smooth; (b) buckyball; (c) deformed; (d) labyrinth. All scale bars in (a–d) correspond to 10 μm. Insets in (a) and (d) show typical high magnification SEM images of the NP surface layers on the NMPs. All scale bars in inset correspond to 100 nm. (e–g) show images of NMPs made from 0.2 g/mL, 0.08 g/mL and 0.02 g/mL polystyrene/DCM concentrations, respectively. All scale bars in (e–g) correspond to100 μm.

**Fig. 3:**
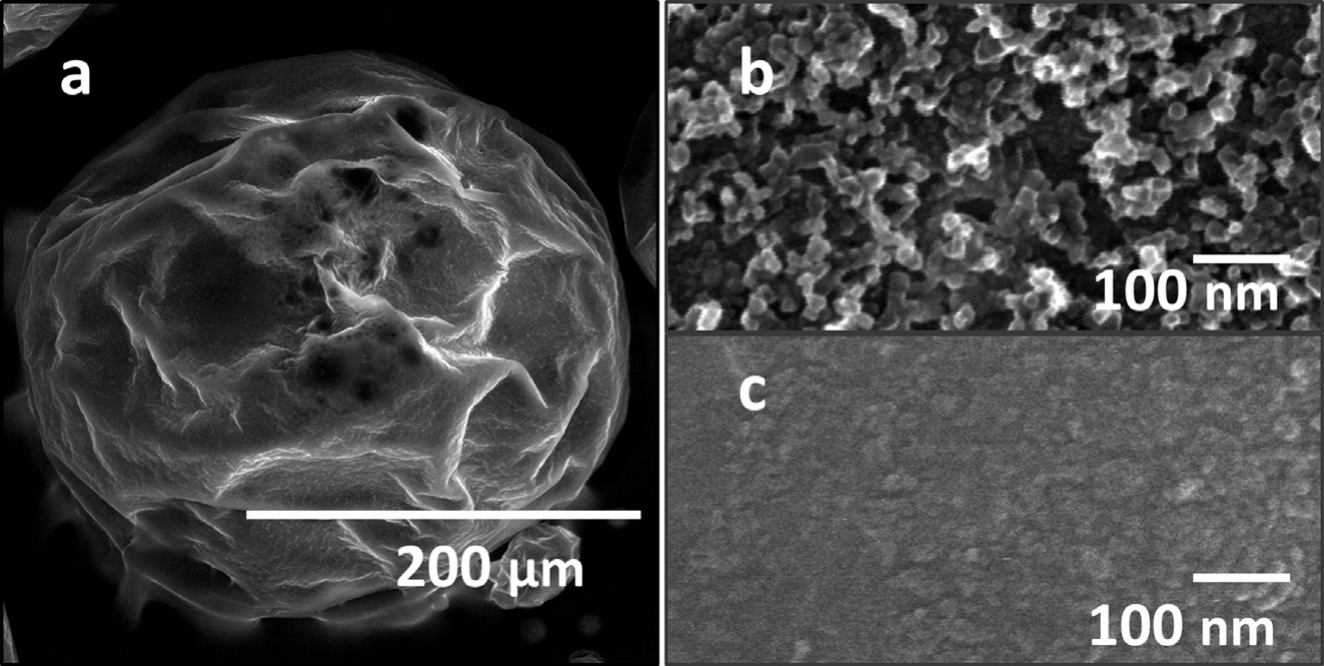
SEM data showing the surface of a magnetic TiO_2_ NMP before (a) and after sonication (b). All scale bars correspond to 100 nm.

**Fig. 4:**
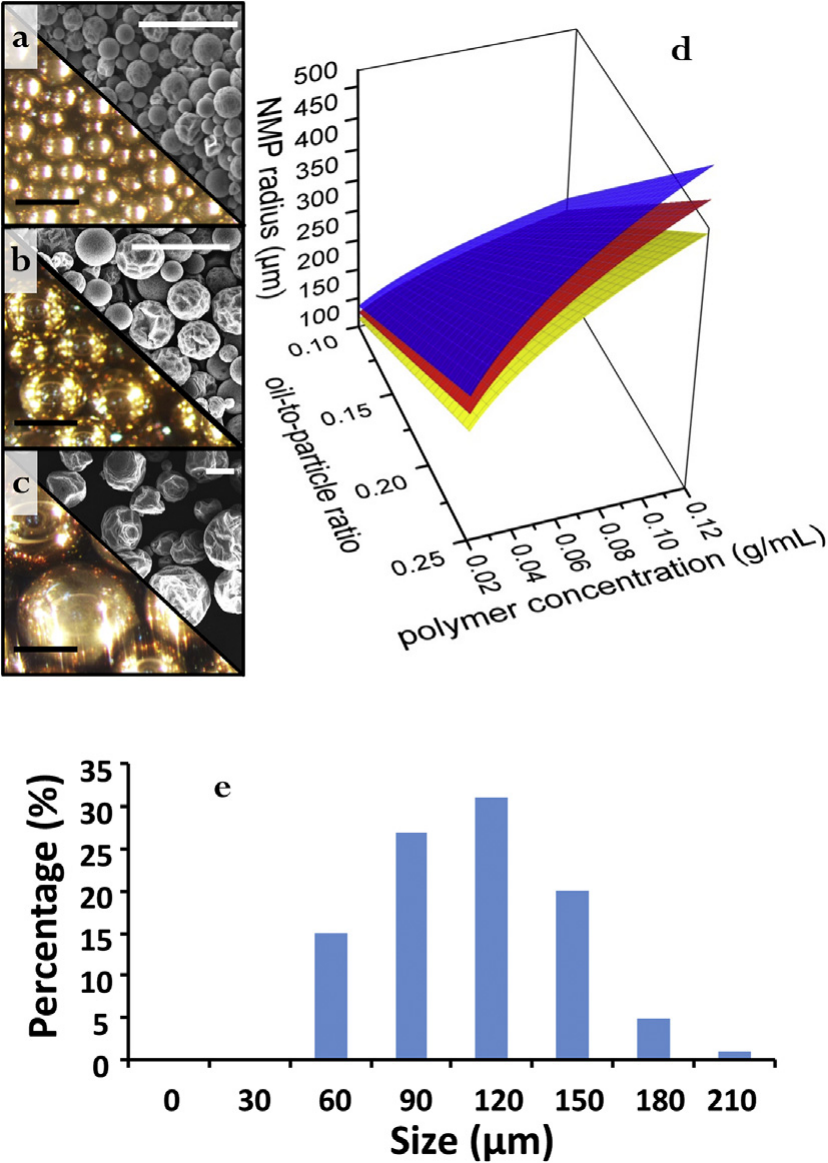
Optical images of emulsions made from different colloid-oil ratios: 5:1 (a), 5:1.5 (b) and 5:2 (c) and corresponding SEM data for resulting NMPs. All scale bars in optical images correspond to 500 μm. All scale bars in SEM images correspond to 1000 μm. 3D surface plots illustrating the effect of polymer concentration and colloid-oil ratio on the final size of NMPs (d). Actual size distribution data of Au NMPs prepared using a typical colloid-oil ratio 5:1 measured via SEM (e).

Fig. 1 compares the hydrophobicity of a surface composed of different types of densely packed NPs as well as the same type of NP but with different surface-cappings. Table 1 presents a list of capping agents along with the corresponding amounts required for modifying the surface of Au NPs to achieve the correct hydrophobicity to generate stable Pickering emulsions. Fig. 2 show SEM images of NMPs with different morphologies obtained by tuning the polystyrene concentration. Fig. 3 presents SEM data of the surface morphology of NMPs before and after removal of the NP layer on the surface. A mathematical model and supporting mathematical data is provided in Fig. 4 to illustrate the effect of experimental parameters on the size of product NMPs.

**Table 1:** List of surface capping agents found to produce a suitable contact angle on Au NPs to generate stable Pickering emulsions.

Chemical name	Chemical structure
3-Mercaptopropionic acid (MPA)	
Thioglycolic acid (TGA)	
2-Mercapto-5-benzimidazolesulfonic acid sodium salt (MBS)	
3-Mercapto-1-propanesulfonate (MPS)	
Polyvinylpyrrolidone (PVP)

## 2 Experimental design, materials and methods

### 2.1 Surface hydrophobicity data for NMPs

Contact angle measurements were performed on surface exposed nanoparticle sheets (SENS) fabricated following a method previously reported by our group [2], [3]. SENSs are formed by fixing a monolayer 2D NP array assembled using promoters at the liquid-liquid interface (LLI) onto a thin polymer sheet in-situ through solvent evaporation. Therefore, SENSs have the same micro-structure as NMPs which makes them perfect candidates for contact angle measurements [1]. As shown in the contact angle data, citrate-capped NPs (θ = 75°) were notably more hydrophobic than PVP-capped NPs (θ = 38°) which generated stable Pickering emulsions.

A full list of hydrophilic capping agents capable of generating stable Pickering emulsions with the aid of promoters tested in this work is shown in Table 1.

Calculations of the amount of molecular capping needed for Au particle surface modification:

The size of Au particles as determined by SEM images varied from 28 nm to 34 nm, therefore the average size of the Au particles is 30 nm in diameter. The surface occupied by one modifier molecule is 0.20 nm^2^ [4].

The amount of Au used is:

$$=\frac{\text{Molecular}\;\text{mass}\;\text{of}\;\text{Au}\times \text{Mass}\;\text{of}\;\text{Au}\;\text{chloride}}{\text{Molecular}\;\text{mass}\;\text{of}\;\text{Au}\;\text{chloride}}=\frac{197\times 0.005}{394}=0.0025\;\text{g}$$

The volume of one Au NP is:

$$=\frac{4}{3}\pi {\text{R}}^{3}=\frac{4}{3}\pi 15^{3}=1.41\times 10^{4}{\;\text{nm}}^{3}$$

The mass of one Au NP is:

$$=\rho V=\left(19.30\times 10^{-21}\right)\times \left(1.41\times 10^{4}\right)=2.73\times 10^{-16}\;\text{g}$$

The number of Au NP in 5 mL colloid is:

$$=\frac{\text{Amount}\;\text{of}\;\text{Au}\;\text{used}}{\text{Mass}\;\text{of}\;\text{one}\;\text{Au}\;\text{NP}}=\frac{0.0025}{2.73^{-16}}=9.16\times 10^{12}$$

The surface area of one Au NP is:

$$=4\pi {\text{R}}^{2}=4\pi 15^{2}=2827.43{\;\text{nm}}^{2}$$

The total surface area in 5 mL colloid is:

=NumberofAuNP×surfaceareaofoneNP

$$=9.16^{12}\times 2827.43=2.59^{16}{\;\text{nm}}^{2}$$

The amount of modifier required for surface functionalization is:

$$=\frac{\text{Total}\;\text{surface}\;\text{area}\;\text{in}\;5\;\text{mL}\;\text{Au}\;\text{colloid}}{\text{Surface}\;\text{area}\;\text{of}\;\text{one}\;\text{modifier}\;\text{molecule}}$$

$$=\frac{2.59\times 10^{16}}{0.20}=1.30^{17}=2.16\times 10^{-7}\;\text{mol}$$

The amount of modifier used is:

=Concentrationofmodifier×volumeofmodifier

$$=10^{-2}\times \;10^{-4}=1\times 10^{-6}\;\text{mol}$$

*ρ*, *V*, *R* refer to density, volume and radius, respectively.

### 2.2 SEM data of NMPs

As shown in the SEM data in Fig. 2a–d, the morphology of the NMPs becomes more and more irregular as the concentration of the polystyrene decreases. Fig. 2e and f shows the relative population of deformed NMPs increasing with decreasing concentrations of polystyrene.

As shown in Fig. 3a, prior to sonication, a densely packed layer of TiO_2_ NPs are present on the surface of the NMP. After extensive sonication, the NPs on the surface are removed to show the bare and smooth surface of the micro-polymer core.

### 2.3 NMP size distribution data

Fig. 4a–c compares the size of the parent Pickering emulsions with their corresponding NMP products. A surface plot illustrating the effect of polymer concentration and colloid-to-oil ratio is shown in Fig. 4d. Within the plot, the lower surface (yellow) represents ideal smooth and spherical NMPs and the offset surfaces (red and blue) represent the effect of different extents of wrinkling to the final diameter of the NMPs. In general, the data shows that when the effects of wrinkling and buckling are taken into account, the predicted size of NMPs becomes larger. Detail mathematical data correlating to the size of product NMPs versus colloid-oil ratio and polystyrene concentration is shown below:

Assuming the number of Au particle is *n*, the volume of the oil is *v* μm^3^, the number of the emulsion droplets is *a*, the concentration of the polymer in the oil is *c* g/mL (*b* g/μm^3^), the radius of one emulsion droplet is *r* μm, the radius of the NMPs is *R* μm. Assuming the Au NPs are spherical, and the diameter of the Au particles *d* is 0.03 μm. The density of polystyrene ρ is 1.04 g/mL (1.04 × 10^−12^ g/μm^3^).

The surface area of an Au NP is:

$$=\frac{\pi d^{2}}{4}\;{\text{μm}}^{2}$$

The whole surface area of all Au NPs is:

$$=\frac{\pi d^{2}}{4}n\;{\text{μm}}^{2}$$

The volume of one emulsion droplet is:

$$=\frac{v}{a}\;{\text{μm}}^{3}$$

The radius of one emulsion droplet is:

$$={\frac{3v}{4\pi a}}^{\frac{1}{3}}\text{μm}$$

The surface area of one emulsion droplet is:

$$=4\pi {\text{r}}^{2}=4\pi {\left(\frac{3v}{4\pi a}\right)}^{\frac{2}{3}}\;{\text{μm}}^{2}$$

The surface area of the all emulsion droplets equals to the surface area of all Au NPs:

$$4\pi {\text{r}}^{2}a=4\pi a{\left(\frac{3v}{4\pi a}\right)}^{\frac{2}{3}}=\frac{\pi d^{2}}{4}n$$

From these equation one derives the volume of one emulsion droplet:

$$\frac{v}{a}=\frac{d^{3}}{233^{0.5}}{\left(\frac{n}{a}\right)}^{1.5}=\frac{233}{d^{6}}{\left(\frac{v}{n}\right)}^{3}\;\text{μ}m^{3}$$

When $v=10^{12}\;{\text{μm}}^{3}$, $n=10\times 10^{12}$, the radius of the emulsion droplet *R* is calculated to be 0.42 mm, which is close to the experimental value 0.25–0.4 mm.

The mass of polystyrene in one emulsion droplet is:

$$\frac{vb}{a}=\frac{233}{d^{6}}{\left(\frac{v}{n}\right)}^{3}b\;\text{g}$$

The volume of polystyrene in one emulsion droplet is:

$${=2.24\frac{10^{14}}{d^{6}}\left(\frac{v}{n}\right)}^{3}b\;\text{μ}m^{3}$$

The radius of the NMP is:

$$=\frac{37677}{d^{2}}\frac{v}{n}\sqrt[{3}]{b}\;\text{μ}m$$

$$=\frac{3.77}{d^{2}}\frac{v}{n}\sqrt[{3}]{c}\;\text{μ}m$$

When $v=10^{12}\;{\text{μm}}^{3}$, $n=10\times 10^{12}$, the radius of the emulsion droplet *R* is calculated to be 180 μm, which is quite different from the observed value 50 μm.
